# Real-Time Dissecting the Entry and Intracellular Dynamics of Single Reovirus Particle

**DOI:** 10.3389/fmicb.2018.02797

**Published:** 2018-11-20

**Authors:** Jia Liu, Cong Yu, Jian-Fang Gui, Dai-Wen Pang, Qi-Ya Zhang

**Affiliations:** ^1^State Key Laboratory of Freshwater Ecology and Biotechnology, Institute of Hydrobiology, Chinese Academy of Sciences, Wuhan, China; ^2^Key Laboratory of Analytical Chemistry for Biology and Medicine (Ministry of Education), State Key Laboratory of Virology, College of Chemistry and Molecular Sciences, Institute of Advanced Studies, Wuhan University, Wuhan, China

**Keywords:** non-enveloped virus, reovirus, entry, real-time, single-particle tracking, clathrin, cytoskeletons, endosomes

## Abstract

Reoviruses are non-enveloped viruses with wide host range, can cause serious infections in animals, plants and microorganism, e.g., aquareovirus, which is capable of causing serious haemorrhagic in aquatic animals. To date, the entry process of aquareovirus infection remains obscure. Real-time single-virus tracking are effective tools for exploring the details in viral infection process, which are crucial for understanding the pathogenic mechanism. Here, we used quantum dots-based single particle tracking technology combined with biochemical assays and ultrastructural observation to reveal unobservable infection steps and map dynamic interactions between a reovirus, *Scophthalmus maximus* reovirus (SMReV), and its host cell in real time. The results showed that the single membrane-bound reovirus particle can enter into the cell within several seconds through nascent clathrin-caoted pits, and most of the particles could internalize into cytoplasm within 30 min post-infection. The specific inhibitors analysis also showed that entry of SMREV depended on clathrin-mediated endocytosis rather than cavolin-mediated endocytosis. The motion analysis of internalized single particle indicated that the reovirus initially experienced slow and directed motion in the actin-enriched cell periphery, while it underwent relatively faster and directed movement toward the cell interior, suggesting that transport of SMReV was dependent on the cytoskeleton. Further, dual-labeling of virus and cytoskeleton and inhibitor analysis both demonstrated that transport of internalized SMReV was firstly dependent on actin filaments at the cell periphery, and then on microtubules toward the cell interior. Then visualization of SMReV trafficking in the endosomes revealed that the internalized reovirus particles were sorted from early endosomes to late endosomes, then part of them were delivered to lysosome. This study for the first time revealed the entry pathway, intracellular dynamic and the infection fate of fish reovirus in host cell in real time and *in situ*, which provided new insights into the infection mechanism of non-enveloped viruses.

## Introduction

Reoviruses were ubiquitous with wide host range. They can cause serious infections in animals, plants, and microorganism and were recognized as important pathogens (King et al., 2011). As a member of the *Reoviridae* family, viruses in *Aquareovirus* can cause sevrious infections in various aquatic animals, and represent important viral pathogens in aquatic animals. It could even infect together with some other viruses, e.g., iridoviruses, rhabdovirus, and herpesvirus, causing serious threat to aquaculture (Zhang et al., 2004; Zhang and Gui, 2012). World aquaculture is the fastest growing food-producing sector in the world (Cressey, 2009), which greatly contributes to food security and poverty alleviation (Naylor et al., 2000; Tilman and Clark, 2014; Costello et al., 2016; Fisheries, 2016). Over the last decade, more and more aquareoviruses have been isolated and completely sequenced (>16 strains) in worldwide, such as *Scophthalmus maximus* reovirus (SMReV) and grass carp reovirus 109 strain (Ke et al., 2011; Chen et al., 2015; Zhang and Gui, 2015), posing a global threat to aquaculture.

Aquareovirus are non-enveloped virus surrounded by a double-layered capsid containing 11 segments (S1–11) of linear double stranded RNA. The genome encode 7 structural proteins (VP1–VP7) and 5 nonstructural proteins. The outer-capsid proteins of reovirus are responsible for initiating infection, stimulate the host immune system and the acid-activated penetration (Liemann et al., 2002; Danthi et al., 2010; Liu J. et al., 2016). The inner capsid proteins possess the enzymatic activities necessary for viral transcription (Odegard et al., 2004). The non-structural proteins are also crucial for viral propagation (Ke et al., 2013). However, the infection mechanism and pathogenesis of aquareovirus were limited characterized.

Virus entry into host cells is the first step of infection and a crucial target for antiviral drug and therapeutic intervention. To establish successful infection, viruses must developed strategies to overcome the membrane barriers. Enveloped viruses can achieve this through membrane fusion mediated by enveloped glycoproteins or endocytosis, such as human immunodeficiency virus (HIV) and influenza (Blumenthal et al., 2012; Sun et al., 2017). Non-enveloped viruses are unable to take advantage of membrane fusion to enter cells and generally apply the endocytosis pathway (Elkin et al., 2016). Several previous studies suggested that mammalian orthoreovirus uses multiple endocytic pathways for cell entry, even a particular preferred pathway by any specific viral strain (Schulz et al., 2012). For instance, four strains of mammalian orthoreovirus were tested in MA104 cells, three showed a dependence on clathrin-mediated endocytosis, while the other one used cavoelin-dependent endocytosis (Gutiérrez et al., 2010; Abdelhakim et al., 2014), which was also required in the entry of avian reovirus (Huang et al., 2011). Upon internalization, the transport to late endosomes is required for yielding a productive reovirus infections (Mainou et al., 2013). In addition, it has been proposed that cytoskeletons are involved in reovirus entry process as the inhibition by cytoskeleton disrupting agents affect the virus infectivity (Sharpe et al., 1982). For the aquareovirus, although the recent two independent studies have demonstrated the endocytosis of the grass carp reovirus (GCRV) in host cells through different methods, one showed GCRV strain 104 enter CIK cells via clathrin-mediated endocytosis (Wang et al., 2018), while another revealed GCRV strain 873 use caveolae/raft-mediated endocytosis as the primary entry pathway to initiate infection in CIK (Zhang F. et al., 2018). So further work is needed to better understand the uptake and intracellular dynamics of aquareovirus. Although some critical entry steps have been investigated based on the use of siRNAs, dominant-negative mutants and chemical inhibitors, a high temporal and spatial resolutions comprehensive picture of reovirus entry process remain to be depicted.

Single particle tracking in living cell not only contribute to monitoring dynamic virus-host cell interactions at the single-virus level, but also facilitate for elucidating infection mechanism by providing *in situ* and real-time evidences (Liu S. L. et al., 2016). In recent years, owing to the superior brightness and stability of quantum dots (QDs), QDs-based single particle tracking has been extensively used for revealing enveloped-virus infection process in living cells, such as influenza, pseudorabies virus, and infectious hematopoietic necrosis virus (Liu et al., 2011; Rosenthal et al., 2011; Zhang Y. et al., 2013; Chu et al., 2014; Pan et al., 2014; Wegner and Hildebrandt, 2015; Li et al., 2017), while there is few about non-enveloped virus tracking (Joo et al., 2011; Zhang F. et al., 2018), which supervise and urge us to challenge to develop convenient tactic for viral tracking and further revealing the entry pathway and the infection journey of the non-enveloped virus, such as aquareovirus, the major pathogens associated with severe hemorrhagic disease in aquaculture animals.

In this study, we elucidated the details in infection process of a non-enveloped virus, SMReV, in the single-particle level through QDs-based single particle tracking combined with biochemical approaches and ultrastructural observation. The entry pathway and intracellular dynamics of single SMReV article were monitored contributing to a better understanding of viral life cycle and pathogenic mechanism.

## Materials and methods

### Cells and virus

The grass carp fins (GCF) cell line used in this study were cultured in TC199 medium supplemented with 10% fetal bovine serum (FBS) at 25°C. For living or fixed cell fluorescence imaging, GCF cells were cultured in 35-mm glass-bottom culture dishes for 16 h to achieve 75% confluence. For biochemical analysis GCF cells were seeded on coverslips in 6-well plates to achieve 75% confluence. SMReV was propagated in GCF cells at 20°C, and virus stocks were kept at −80°C in our laboratory.

### Preparation of plasmids and antibodies

For preparing the antibodies, two recombinant expression plasmids pET-32a-VP7 and pET-32a-NS25 were produced, respectively, as the previous method (Ke et al., 2013). In brief, the total RNA was extracted from the SMReV infected cells using TRIzol reagent (Invitrogen). The viral outer capsid protein VP7 and non-structural protein NS25 encoding gene (S10 and S11), were amplified from the total RNA obtained above by reverse transcription PCR (RT-PCR) with the primer VP7-F/R and NS25-F/R, respectively (Table 1). Then, the RT-PCR products were digested with *Kpn* I and *EcoR* I enzymes and then ligated into vector pET-32a, resulting in pET-32a-VP7 and pET-32a-NS25 construct. To facilitate early endosomes detection, pRFP-Rab5 and pRFP-Rab7 were constructed. Using total RNA extracted from fish cells as template, fish Rab5 and Rab7 encoding genes were amplified by RT-PCR using the designed primers Rab5-F/R and Rab7-F/R (Table 1), and The PCR products were inserted into pRFP-C1 vector, resulting in pRFP-Rab5 and pRFP-Rab7 constructs.

**Table 1:** The primers used in this study (enzyme cleavage site was underlined).

**Primer**	**Primer sequences (5′-3′)**	**Gene (GenBank sequence)**	**Plasmid construction or analysis**
VP7-F	gtaggtaccatggagaccaaaccaattcttccaac (*Kpn* I)	S10 (HM989939)	pET-32a-VP7
VP7-R	ccggaattcatcctcacccacaggcgcg (*EcoR* I)		
NS25-F	ggagaattcatggctcaggacttga (*EcoR* I)	S11 (HM989940)	pET-32a-NS25
NS25-R	ctcaagcttctactcaaatacgccc (*Hind* III)		
Rab5-F	ccggaattccatggccaataggggaggagc (*EcoR* I)	Rab5 (NM_201485)	pRFP-Rab5
Rab5-R	cgcggatccttagttgctgcagcaggggg (*BamH* I)		
Rab7-F	ccggaattccatgacatcaaggaagaaagt (*EcoR* I)	Rab7 (NM_200928)	pRFP-Rab7
Rab7-R	cgcggatcctcagcagctacaggtctctg (*BamH* I)		
S8-F	ggctgaagtttgatgctatgtggc	S8 (HM989937)	Real-time quantitative PCR
S8-R	ggtagacttgggcttgaatagacacg		

For preparing the anti-VP7 antibody, the recombinant plasmid pET-32a-VP7 was transformed into an *E. Coli* strain BL21 (DE3) for protein expression. Then the fusion protein was purified and used to immunize mice as previously reported (Liu J. et al., 2016). The experiment were approved and performed in accordance with the guidelines of the Institutional Animal Care and Use Committee of the Institute of Hydrobiology, Chinese Academy of Sciences. The anti-NS25 antibody was also prepared in the same way.

### Purification and biotinylation of SMRev

SMReV were propagated in GCF cells and harvested at 7 days postinfection (p.i.), and the cell debris was removed by centrifugation at 4°C. The supernatant was used for virus biotinylation, as below: roughly 300 ml virus supernatant (approximately 10^7^ copies/ml) was reacted with 300 μl Sulfo-NHS-LC-Biotin (0.01 mg/μl, Thermo) at room temperature for 2 h. Then, the supernatant of biotinylated virus (Bio-SMReV) and unbiotinylated (SMReV), respectively, was purified by ultracentrifugation and sucrose discontinuous gradient (20, 30, 40, and 50%) centrifugation at 110,000 g for 1.5 h as previously reported (Ke et al., 2011). During this process, the unbound biotin and impurities were removed. The purified Bio-SMReV and SMReV particles were obtained and stored at −20°C until use.

### Labeling of SMRev with QDs

The non-enveloped RNA virus, SMReV, was labeled by the biotin–streptavidin interaction to link biotinylated SMReV with streptavidin-modified QDs (SA-QDs). The method consisted of standard procedures: 100 μl Bio-SMReV were bound to monolayer of GCF cells for 30 min at 4°C and then washed thrice with pre-chilled PBS contained with 0.1% bovine serum albumin (BSA). The plates were then incubated with 100 μl 5 nM streptavidin-modified QDs (SA-QDs 605/705, Invitrogen/Wuhan Jiayuan) at 4°C for 15 min. After three additional washes with PBS (with 0.1% BSA), the QDs labeled SMReV (QDs-SMReV) particles were obtained and could be used for subsequent analysis.

### Immunofluorescence assay (IFA) and infectivity analysis

For analysis of labeling efficiency, the purified Bio-SMReV or SMReV was added to the monolayer GCF cells, respectively. The viruses were labeled by SA-QDs as above; then the QDs-SFTSV was immunostained by IFA. The infected GCF cells were fixed with 4% paraformaldehyde, and blocked with 2% BSA. After washed thrice with PBS, cells were incubated with anti-VP7 antibody diluted (1:300) in 1% BSA at room temperature for 2 h. After washing, Dylight 488 conjugated goat anti-mouse IgG (Dylight 488, Abbkine) was added and incubated at room temperature for 2 h, obtained the anti-VP7-Dylight 488 labeled virus. The cells were washed and imaged by confocal laser scanning microscopy (CLSM) with two kind of fluorescence signals, and colocalized efficiency of the two kinds of signals was calculated as labeling efficiency. To visualize the internalization of Transferrin, GCF cells grown on coverslips were plated on ice for 10 min and then incubated with Alexa Fluor™ 568 conjugate Human Transferrin (Themo) at 25 μg/ml for 30 min at 25°C. The non-internalized transferrin was removed by citrate buffer (40 mM sodium citrate, 10 mM KCl, and 135 mM NaCl at pH 3.1). The cells were imaged by confocal laser scanning microscopy.

For the infectivity analysis of labeled virus, GCF cells were incubated with 100 μl of purified SMReV, Bio-SMReV or QDs-SMReV (100 μl bio-SFTSV labeled with QDs), and the infection cells were harvested at 48 h postinfection. Viral infectivity were determined by Real-time qPCR as follows. Three independent experiments were performed.

### Real-time qPCR

Total RNA were extracted from GCF cells treated or untreated with inhibitors, respectively. Next, cDNA was synthesized using M-MLV reverse transcriptase (Promega). For real-time qPCR, the viral gene S8 was detected by the primer S8-F/R (Table 1) and the β-actin was used as an internal control with primer. Each sample contained a 25 μl reaction system as the instructions of SYBR green real-time PCR Master Mix (Toyobo). The cycling conditions were as follows: an initial hold at 94°C for 10 min, followed by 40 cycles consisting of 94°C for 15 s, 60°C for 1 min. Following amplification, DNA melting curve analysis was performed to confirm the specificity of the PCR products. Triplicate independent experiments, three duplicates for each sample, were performed. SMReV gene transcription level was quantified as the percentage of SMReV RNA copies of Bio-SMReV or QDs-SMReV infected cells relative to SMReV infected cells as the y-axis. The data represented the mean values and standard deviations of the results from independent experiments. The infectivity of virus in untreated cells was factitiously set as 100%.

### Labeling of cellular components

To label the cell membrane and lysosome, the cells were incubated with CellMask™ and LysoTracker (Invitrogen), respectively, according to the manufacturer's instructions. To facilitate other cellular components detection, fluorescent protein (RFP/ GFP) fusion proteins were expressed. Different cellular components, clathrin, caveolin, early endosomes, late endosomes, actin filaments, and microtubule were sequentially labeled with pEGFP-LCa, pEGFP-Cav, pRFP-Rab5, pRFP-Rab7, pEGFP-LifeAct, and pEGFP-MAP4 (Table 2). The plasmids were transfected into cultured GCF cells with Lipofectamine 2000 according to the manufacturer's protocols. After another 36 h of culture, cells were infected with QDs-SMReV for fluorescence observation.

**Table 2:** The corresponding labeling fluorescent tags/dyes for SMReV and cellular components used in this study.

**Virus or cellular components**	**Fluorescent labels**	**Laser (nm)**	**Filter (nm)**
**VIRUS**			
SMReV particles	QDs 605	561	605–620
SMReV particles	QDs 705	488	685–740
Anti-VP7 of SMReV	Dylight488 conjugated goat anti-mouse IgG	488	525–550
**CELLULAR COMPONENTS**			
Cytomembrane	CellMask™	640	685–740
Clathrin	pEGFP-LC	488	525–550
Caveolin	pEGFP-Cav	488	525–550
Microtubule	pEGFP-MAP4	488	525–550
Actin filament	pEGFP-LifeAct	488	525–550
Lysosomes	Lyso-Tracker	488	525–550
Early endosomes	pRFP-Rab5	561	605–620
Late endosomes	pRFP-Rab7	561	605–620

### Inhibition of SMRev infection

Several chemically inhibitors were used to block different viral cellular entry and intracellular transport pathway. Here, chlorpromazine (CPZ, 0–35 μM) and sucrose (0–300 mM) for inhibiting clathrin-mediated endocytosis, filipin III (0–50 μg/ml) and nystatin (0–25 μg/ml) for inhibiting caveola-mediated endocytosis, cytochalasin D (CD, 0–20 μM) for blocking actin filament elongation at the barbed end, nocodazole (0–10 μM) for disrupting the microtubule, NH_4_Cl (0–7.5 mM) and chloroquine (CQ, 0–75 μM) for disrupting the acidification were applied. The monolayer of GCF cells was treated with each inhibitor for 1.5 h prior to SMReV infection. The inhibitor-treated cells were infected with SMReV for 30 min (fluorescent observation) or 2 h (biochemical assays) at 25°C in the continued presence of the inhibitors, while cells treated with PBS or dimethylsulfoxide (DMSO) were used as negative controls. Then cells were washed once with citrate buffer to remove non-internalized viruses and washed thrice with PBS, then fixed with 4% paraformaldehyde for fluorescent observation or harvested at 72 h p.i. to prepare for real-time qPCR as above description or western blotting analysis. For real-time qPCR analysis, triplicate independent experiments, three duplicates for each sample, were performed. SMReV gene transcription level was quantified as the percentage of SMReV RNA copies of inhibitor-treated cells relative to untreated control cells as the y-axis. The data represented the mean values and standard deviations of the results from independent experiments. The concentrations of inhibitor were used as the x-axis. The cytotoxic effects to the cells of the inhibitors were determined using cell counting kit-8 (Dojindo). It was suggested that inhibitors treatment did not cause cell toxicity (date not shown).

### Western blotting analysis

The protein expression was tested with western blotting as described previously (Liu J. et al., 2016). The anti-NS25 antibody acted as the primer antibody, at a dilution of 1:500, and alkaline phosphatase (AP)-coupled goat anti-mouse IgG was used as the secondary antibody (Promega). Internal control reactions to detect β-actin were carried out simultaneously. Band were visualized using alkaline phosphatase (AP) substrate solution. The brightness of the bands reflected the protein expression levels.

### Live cell fluorescence imaging and analysis

For single-color tracking experiments, cells incubated with QDs labeled SMReV (as above description) were observed by CLSM, immediately. For dual-color observation, specific cellular components were labeled as described above prior to incubation with QDs-SMReV. All observations were performed at 25°C. Fluorescent images were obtained through a spinning-disk confocal microscope (Andor Revolution XD) with an on-line culture system (INUBG2-PI) and an EMCCD (Andor iXon DV885K) under a 100 × objective. The corresponding laser and filter were selected for different fluorescent labels imaging as Table 2.

To quantify the colocalization extent of two fluorescent signals, intensity correlation analysis (ICA) was performed by using WCIF Image J. tMr, tMg, and intensity correlation quotient (ICQ) values were calculated from 20 cells from triplicate independent experiments. tMr indicates the percentage of the red signals colocalized with green signals in the image. tMg indicates the percentage of the green signals co-localized with red signals in the image. ICQ, ranging from −0.5 (independent signals) to +0.5 (dependent signals), could be used for comparation statistically (+0.1 to +0.5 implies a strong covariance) (Liu et al., 2012). To construct 3D confocal image of the cells incubated with QD-SMReV in different times, three-channel Z-stacks were recorded with the gap of 0.3 μm, processed by Andor IQ software and located at the position of interest. The movements of labeled SMReV were tracked and analyzed using Image-Pro Plus 6.0. The trajectories of labeled viruses consisted of the traveling distances between consecutive frames were generated by tracking the representative particles. The time trajectories of the velocity indicated the instantaneous velocity of a virus particle in a living cell. MSD was calculated by an equation using a compiled program based on MATLAB. And the diffusion coefficient (D) and the average speed (V) of the particle movement were further obtained. If the relationship of the mean square displacement (MSD) and the time interval (nΔt) was fitted with an equation of MSD = 4DΔt + (Vt)^2^, the virus particle experienced a directed movement, which indicated the virus likely transported along the cytoskeleton. If the relationship could be fitted with MSD = 4DΔt^α^ (α is a constant <1), the trajectory performed a restricted motion, which implied the virus interacted with its receptor or other organelles. If the plot was fitted with MSD = 4DΔt, it suggested that the particle moved in free diffusion mode (Liu A. A. et al., 2016). The *p*-values were obtained from triplicate independent experiments through SPSS software using ANOVA, and the results were considered statistically significant for *p* < 0.05.

### Electron microscopy

For examination of the morphology of the purified SMReV and Bio-SMReV, the purified virus particles were negatively stained with 2% (w/v) phosphotungstic acid (PTA, pH 6.8) and observed by transmission electron microscope (TEM, JEM-1230), respectively.

Ultrathin sections were performed as described previously (Gao et al., 2015), GCF cells were incubated with SMReV at an MOI of 20 for 30 min at 4°C to allow the virus to binding to the plasma membrane. Cells were then incubated for 0, 15, and 30 min at 20°C. These cells were then fixed in 2% glutaraldehyde in PBS. Ultrathin sections of these cells were examined by electron microscopy.

## Results

### QDs specifically labeling the reovirus

**Figure 1:**
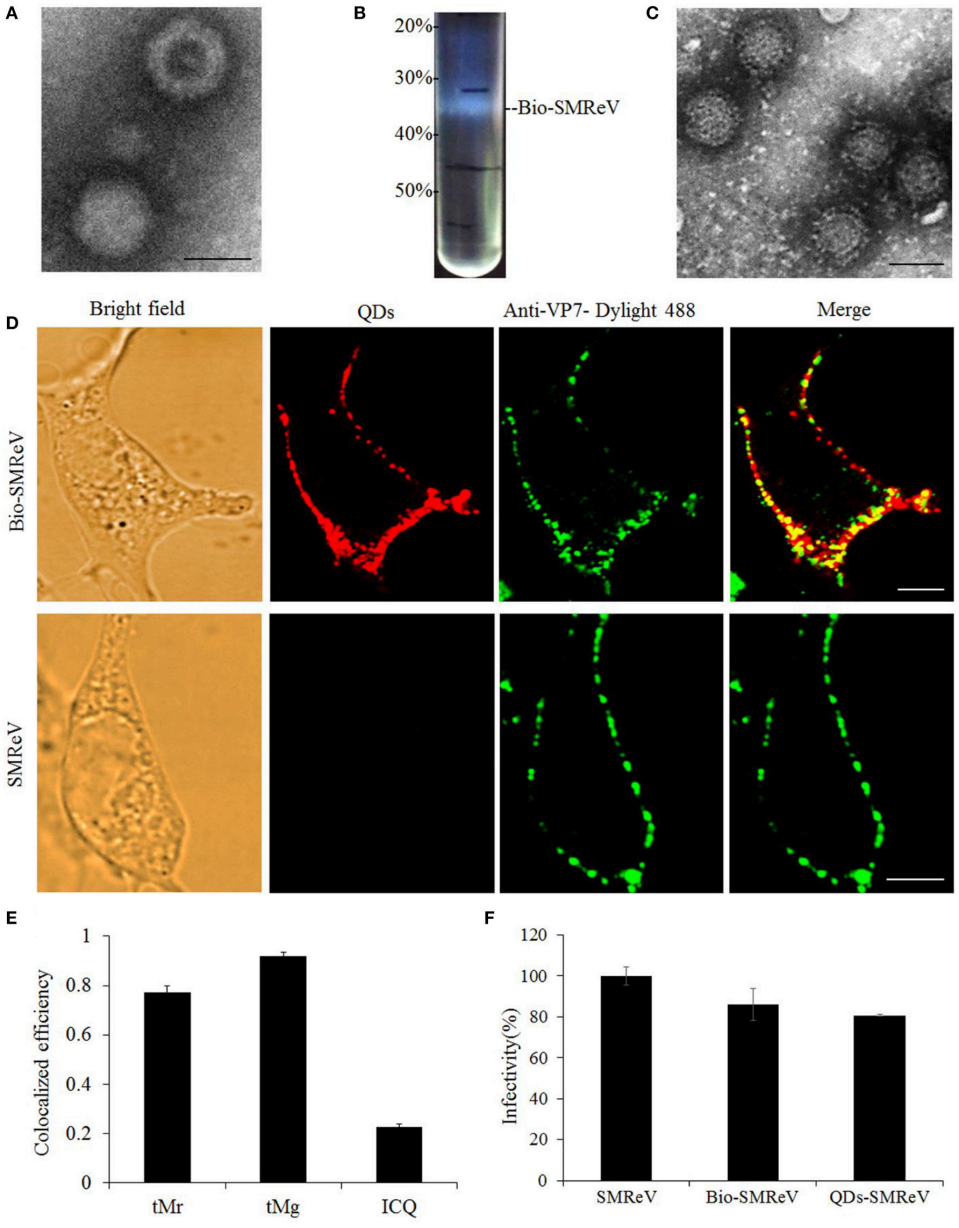
Labeling of SMReV with QDs. **(A)** Electron micrograph of negatively stained purified unbiotinylated SMReV. Bar: 50 nm. **(B)** Image of ultracentrifuge tube after sucrose discontinuous gradient (20, 30, 40, 50%) centrifugation. The high density opalescent virus band (Bio-SMReV) was observed between 30–40% sucrose. **(C)** Electron micrograph of negatively stained purified Bio-SMReV. Bar: 50 nm. **(D,E)** Colocalization analysis of the QDs signals with anti-VP7-Dylight 488 signals. **(D)** Showing adherent cells (Bright field), confocal images of QDs-SMReV (QDs, red) and anti-VP7-Dylight 488 labeled SMReV (green), and the merge image (merge, yellow). Bar: 5 μm. **(E)** Histograms of tMr (the percentage of red signals colocalized with green signals in the images), tMg (the percentage of green signals colocalized with red signals in the images) and intensity correlation quotient (ICQ) values (+0.1–+0.5 implies a strong covariance). **(F)** Infectivity assay. GCF cells (*n* = 20) were infected with SMReV, Bio-SMReV, or QDs-SMReV and collected at 48 h p.i. for viral infectivity analysis by Real-time qPCR.

In order to visualize the course of SMReV entry at the single-virus level in real time, SMReV virions were biotinylated (Bio-SMReV) and purified by ultracentrifugation (Figure 1B). To avoid the damage to the viral particles that may be caused by additional purification steps in the *in vitro* labeling method, Bio-SMReV was bound to the GCF cells surface and incubated with streptavidin-modified QDs *in situ*, then extensive washing with PBS were used to remove unbound virions and QDs (Liu et al., 2012). TEM showed that compared with unbiotinylated SMReV (Figure 1A), the purified Bio-SMReV particles also kept integrated capsids (Figure 1C), indicating that the biotinylated particles still retain the native biological structure. The specificity and efficiency of QDs labeling were examined by analyzing co-localization efficiency of QDs signals (red) with immunofluorescence signals (green) using anti-SMReV VP7 antibody. Under the confocal microscope, the QDs-SMReV exhibited red fluorescence signals around the adherent cells surface (Figure 1D, Bio-SMReV, QDs). Then the QDs-SMReV immunostained with anti-SMReV VP7 antibody exhibited both red and green fluorescence (Figure 1D, Bio-SMReV, Merge). As Figure 1E shown, 78% of the red fluorescence signals were colocalized with the green signals in Bio-SMReV, while 93% of the green signals were colocalized with the red signals, which may be due to the not high efficiency of the uncommercial polyclonal anti-VP7 antibody for immunofluorescence assay. While in the control (unbiotinylated SMReV), the green fluorescence signals were distributed around the cells, but no red fluorescence signal was detectable from the viral particles (Figure 1D, SMReV), indicating that there was no obvious non-specific adsorption between SA-QDs and cells or unbiotinylated SMReV. The infectivity of Bio-SMReV and QDs-SMReV was tested with the parental SMReV as a control; the results showed that neither biotinylation nor conjugation with SA-QDs substantially impaired the infectivity of SMReV (Figure 1F). These indicated that virus particles were efficiently labeled *via* QDs, therefore the red fluorescence signals of QDs could reveal SMReV particles and be suitable for imaging single reovirus particles.

### Tracking reovirus particle passing through the plasma membrane

**Figure 2:**
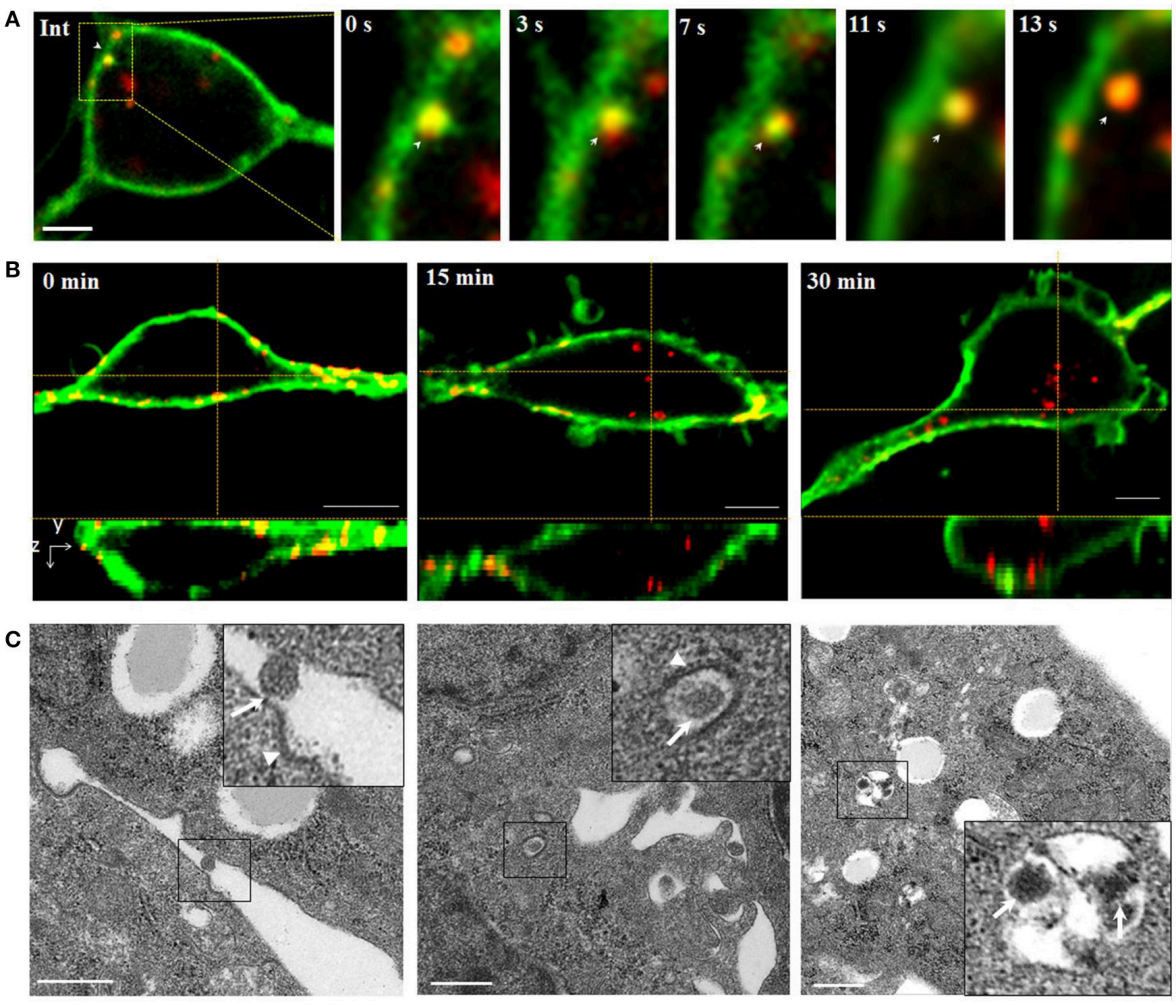
Tracking and imaging of QDs-SMReV entry into living GCF cell. **(A)** Membrane-bound single QDs-SMReV particle (arrows) embedded in and passed through the plasma membrane (green), showing the image (Int) and the enlargement images at different time (0–13 s). Bar: 5 μm. **(B)** Three-dimensional (3D) confocal images of GCF cells (green) at different postinfection time (0–30 min) infected with SMReV. It was shown that SMReV particles attached to plasma membrane (yellow) and then passed through the membrane into cell interior. Bar: 5 μm. **(C)** Ultrastructural micrographs of GCF cells infected with SMReV at various postinfection time (0–30 min). Viral particle was located on the plasma membrane near a nascent depression with shallow plasma membrane indentations at 0 min, contained in a vesicle with a visible cytoplasmic coat at 15 min and included in a lager endosomal vesicles at 30 min. Bar: 500 nm.

Initially, successive snapshots captured single QDs-SMReV particles (*n* = 6) internalizing into living GCF cell visually. The viral particles (red) firstly attached to and embedded into the plasma membrane (green) (Figure 2A, Int). Then the virus invaginated into the plasma membrane, gradually pinched off from the plasma membrane and was trapped into a vesicle (green signal) formed from plasma membrane, finally entered into the cytoplasm within 8–24.3 s (mean 14.8 ± 2.4 s, *n* = 6) (Figure 2A, 0–13 s). The course of multiple SMReV particles entry were also observed. Cells were plated rapidly at 20°C to initiate infection for 0, 15, 30 min after incubated with QDs-SMReV at 4°C for 30min. As Figure 2B shown, the virus particles bound to the cell plasma membrane without entry at 0 min, subsequently, at 15–30 min, particles passed through the plasma membrane and located toward the center of cytoplasm. Further, ultrastructural observation also revealed the infection process. Firstly, at 0 min postinfection, SMReV particles bound to the plasma membrane closely to an ex-existed indentation with electron-dense structures (Figure 2C, left). Then, the internalized SMReV particles was observed within a vesicle identified with shallow plasma membrane indentations with a visible cytoplasmic coat at 15 min postinfection (Figure 2C, middle). At later stage, several SMReV particles were simultaneously located in lager vesicles in the cell interior, which enveloped with smooth single-membrane (Figure 2C, right). These evidently reveled different status of the viral entry process, which were consistent with the above results of the QDs-SMReV. It was indicated that membrane-embedded single virus particle could pass through the membrane within several seconds, and most of SMReV particles could internalize into cytoplasm within 30 min postinfection.

### The reovirus particle enter cells via clathrin-mediated rather than caveola-mediated endocytosis

**Figure 3:**
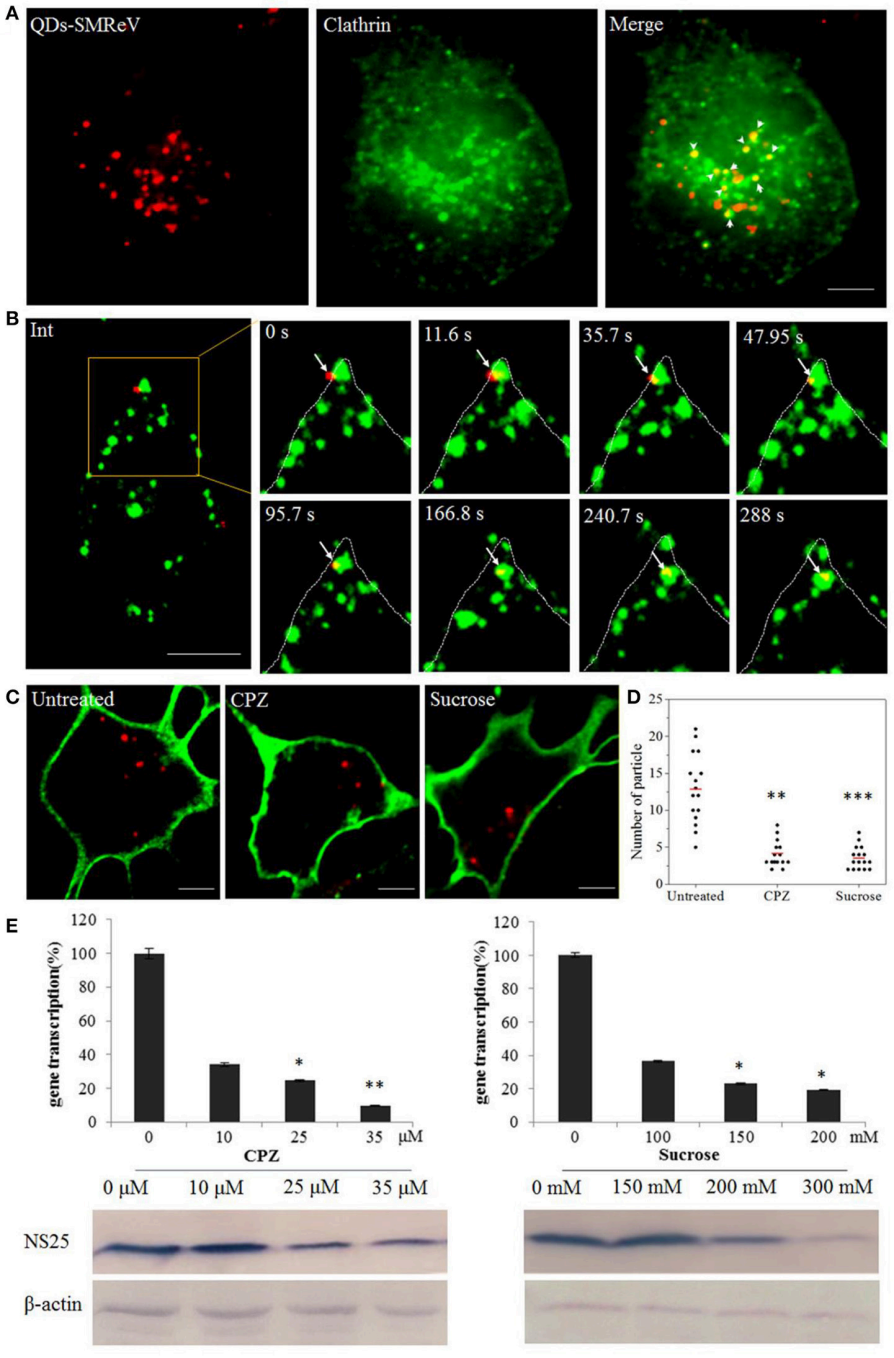
Entry of SMReV into GCF cells is dependent on clathrin-mediated endocytosis. **(A)** Colocalization analysis of SMReV and clathrin through fluorescence images. QDs-SMReV (red) and clathrin (green) labeled by pEGFP-LCa were extensively colocalized (Merge, yellow, arrows mark). Bar: 5 μm. **(B)** Snapshots of a QDs-SMReV particle internalized via nascent CCPs. Sequences of image were captured during the virus internalization at different time point (0–288 s, arrows). Bar: 5 μm. **(C,D)** Uptake of SMReV into the host cells were inhibited by chlorpromazine and sucrose. **(C)** Confocal images of QDs-SMReV (red) infected GCF cell untreated or treated with chlorpromazine (10 μM) or sucrose (200 mM) were obtained at 2 h p.i. The plasma membrane were labeled with CellMask (green). Bar: 5 μm. **(D)** Numbers of internalized QDs-SMReV particles in the cytoplasm of the untreated or treated cells. The bar showed the means of the data (*n* > 15 for each group). **(E)** Inhibitors of clathrin-mediated endocytosis reduced the SMReV infection. GCF cells were untreated (0 μM) or treated with CPZ (10–35 μM, left) or sucrose (150–300 mM, right) for 2 h, infected with SMReV. Then the infected cells were collected at 72 h p.i. for gene transcription levels detection by Real-time qPCR (upper panel) and NS25 protein expression analysis by Western blotting analysis (lower panel). The viral gene transcription of untreated cells was factitiously set as 100%. The results are from triplicate experiments, and error bars indicate the means ± SD. ^*^p < 0.05, ^**^p < 0.01, ^***^p < 0.001. β-actin is used as a loading control.

The major endocytosis pathways include the clathrin-, caveolin-dependent endocytosis and micropinocytosis. To determine whether SMReV entry was associated with clathrin, dual-color fluorescence imaging was subsequently performed to visualize the entry of QDs-SMReV into host cells transiently expressing an enhanced green fluorescent protein fused with clathrin light chain (EGFP-Cla). As Figure 3A shown, 56% QDs-SMReV colocalized with clathrin clusters in GCF cells (*n* = 15) at 30 min postinfection, displaying that the entry of SMReV particles was associated with clathrin-mediated endocytosis. Further, successive snapshots captured the virus particles (*n* = 6) entered into the cytoplasm through clathrin clasters. As Figure 3B and Video S1 shown, initially, particles were bound to a site near a pre-existing clathrin cluster, stepwise moved closer to and coated by the clathrin cluster, and invaginated into the plasma membrane in 35.7 s; following by the clathrin-coat pit containing the particle pinched off from the plasma membrane into the cytoplasm forming the clathrin-coat vesicle from 35.7 to 47.9 s; finally the clathrin-coat vesicle containing QDs-SMReV moved stepwise toward the cell interior in the cytoplasm from 95.7 to 288 s. These revealed that SMReV was internalized into the host cell via nascent clathrin-coat pit.

To further confirm the role of clathrin-mediated endocytosis in the SMReV entry, two well-known inhibitors, CPZ and sucrose, which inhibit the assembly of clathrin-coat pit/clathrin-coat vesicle, were used. The effects of the inhibitors on SMReV infectivity were investigated through QDs labeling combined with biochemical assay, including real-time qPCR and western blotting. The confocal images showed a noticeable reduce in the amount of internalized SMReV particles when the cells treated with CPZ (10 μM) and sucrose (200 mM) compared with controlled cells (Figure 3C). Quantification of SMReV-infected cells (*n* > 15) showed that the number of internalized QDs-SMReV in CPZ or sucrose treated cells was ~3- and 4-fold lower than in untreated cells, respectively (Figure 3D). CPZ and sucrose also significantly inhibited SMReV proliferation, which caused concentration-dependent decreases in the SMReV S8 gene transcription and NS25 protein expression levels, compared with the controlled cell. The level of S8 gene transcription decreased to 33, 22, and 10% of the control in the presence with 10, 25, 35 μM of CPZ, respectively (Figure 3E, left). Western blotting showed that cells pretreatment with CPZ (≥25 μM) leaded to a significantly low expression of NS25 protein. As shown in Figure 3E, right, treatment with 200 mM sucrose also obviously inhibited viral gene transcription and protein expression. To verify the effects of these two drugs on clathrin-mediated endocytosis, we applied Alexa Fluor™ 568 Conjugate Transferrin, which was an endogenous substrate of the clathrin-mediated endocytosis, as a positive control (Cureton et al., 2009). Both CPZ and sucrose could blocked the internalization of Transferrin compared to untreated cells (date not shown), which demonstrates the specific disruption of clathrin-mediated endocytosis by these two drugs. These results indicated that clathrin acted as an essential component for cellular entry of SMReV.

**Figure 4:**
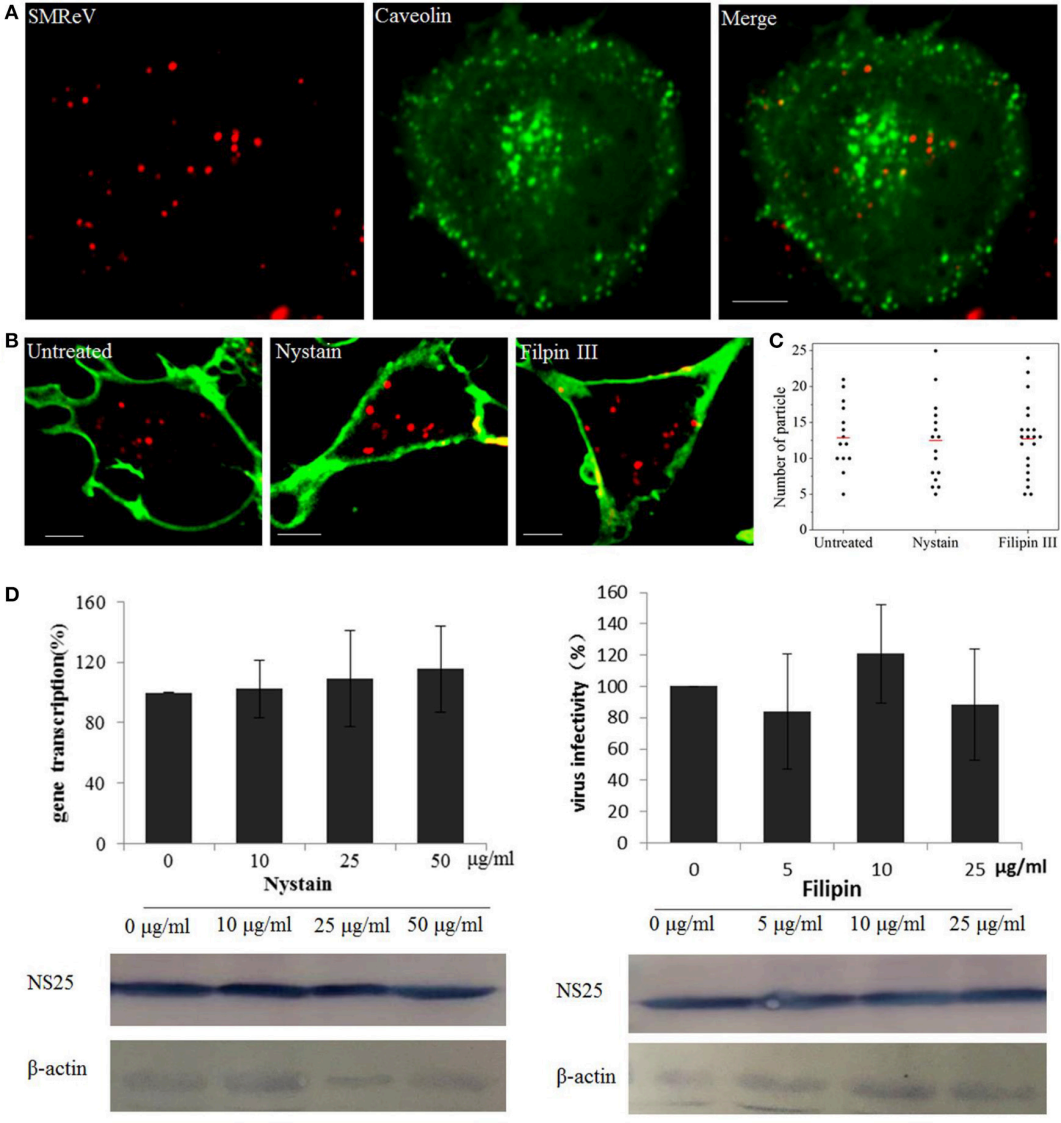
Entry of SMReV into GCF cells is independent on caveolin-independent endocytosis. **(A)** Colocalization analysis of SMReV and caveolae. Fluorescence images of QDs-SMReV (red) and pEGFP-Cav labeling caveolae (green) were captured. The colocalization signals (yellow) were rare. Bar: 5 μm. **(B,C)** Uptake of SMReV into the host cells were not blocked by inhibitors of caveolin-dependent endocytosis. **(B)** Confocal images of QDs-SMReV (red) infected GCF cell untreated or treated with nystatin (10–50 μg/mL, left) or filipin III (0–25 μg/ml, right) were obtained at 2 h p.i. The plasma membrane were labeled with CellMask (green). Bar: 5 μm. **(C)** Numbers of internalized QDs-SMReV particles in the cytoplasm of the untreated or treated cells. The bar showed the means of the data (*n* > 15 for each group). **(D)** Inhibitors of caveolin-mediated endocytosis did not reduce the SMReV infection. GCF cells were untreated (0 μM) or treated with nystatin (10–50 μg/mL, left) or filipin III (0–25 μg/ml, right) for 2 h, infected with SMReV. Then the infected cells were collected at 72 h p.i. for gene transcription levels detection (upper panel) and NS25 protein expression analysis (lower panel). The viral gene transcription of untreated cells was factitiously set as 100%. The results are from triplicate experiments, and error bars indicate the means ± SD. ^*^p < 0.05, ^**^p < 0.01, ^***^p < 0.001. β-actin is used as a loading control.

The caveolae-mediated endocytosis was also assayed with nystatin and Filipin III, two sterol-binding drugs that disrupts the cholesterol-rich caveola-containing membrane by sequestering cholesterol, at different concentrations. Nevertheless, fluorescence images showed only rare colocalization between SMReV and caveolae (Figure 4A). Simultaneously, nystatin and Filipin III could not block the uptake of QDs-SMReV into the cytoplasm (Figures 4B,C). The infectivity analysis also showed that S8 gene transcription and NS25 protein expression had no significant differences between controlled cells and nystatin or Filipin III treated cells (Figure 4D). The data confirmed that entry of SMReV was mediated through clathrin-mediated endocytosis, rather than the caveolae-mediated pathway.

### Directed motion of reovirus along the cytoskeleton

**Figure 5:**
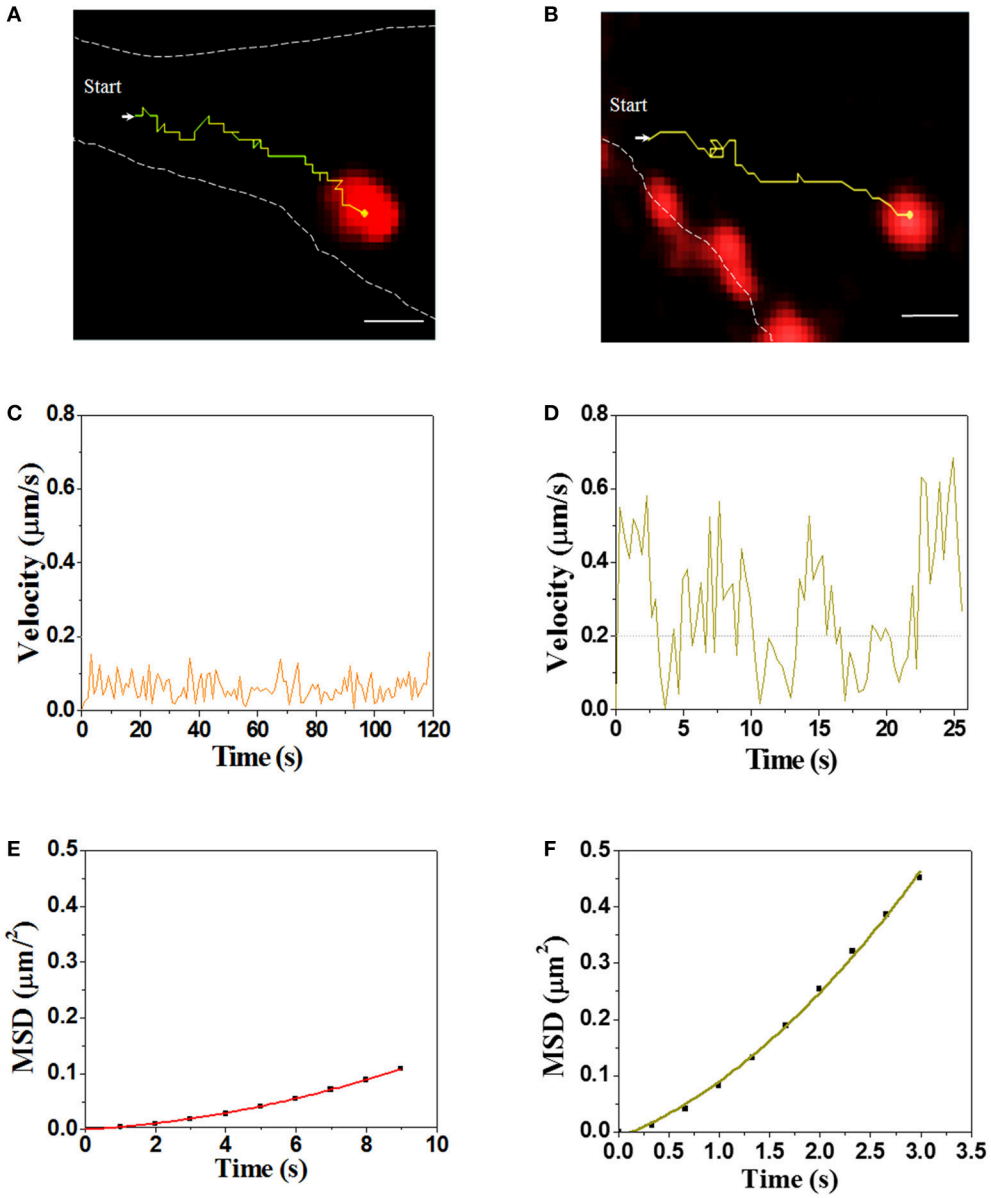
Dynamic of single SMReV particle transport in living GCF cytoplasm. **(A,B)** Tracking single SMReV particles transport in living host cell, showing confocal images of SMReV transported in GCF cytoplasm. Yellow lines indicated trajectories of SMReV particles (red) and the dashed white lines indicated plasma membranes. Bar: 1 μm. **(C,D)** Instantaneous velocities of the virus transport shown in **(A,B)**, respectively. The orange line represented the slow movement of the virus particle in the host cell periphery region, and the yellow line represented the rapid movement of the virus particle in the host cell interior, respectively. **(E,F)** The mean square displacement (MSD)-time plots of the virus particle movement shown in **(C,D)**, respectively.

Real-time visualization allows us to explore unambiguously moving path and dynamic of virus transport in the cytoplasm. Single SMReV particles (*n* = 10) transporting in host cytoplasm were tracked by real-time imaging, and the instantaneous velocity of internalized virus particle were analyzed. As Figure 5 and Videos S2, S3 shown, two trajectories of the virus particles transport in cytoplasm had exhibited the two different motions of SMReV. The initial motility of virus particle was slow (mean 0.06 ± 0.04 μm/s, *n* = 10) in the actin enriched cell periphery (Figure 5C). While upon entering into the cell, the virus particle moved relatively faster (0.23 ± 0.13 μm/s, *n* = 10) toward the cell interior (Figure 5D). And the relationship between MSD and nΔt was fitted to reveal the motion mode, shown as Figures 5E,F. The apparent upward curvature of MSD-nΔt plots was fitting with an equation of MSD = 4DΔt + (Vt)^2^, indicating the virus particle experienced a directed movement dependent on cytoskeleton. And the diffusion coefficient (D) were 0.001 μm^2^/s in the cell periphery, and 0.018 μm^2^/s toward the cell interior along the microtubule. These revealed that the virus particle experienced a directed movement along the cytoskeleton in the cytoplasm.

**Figure 6:**
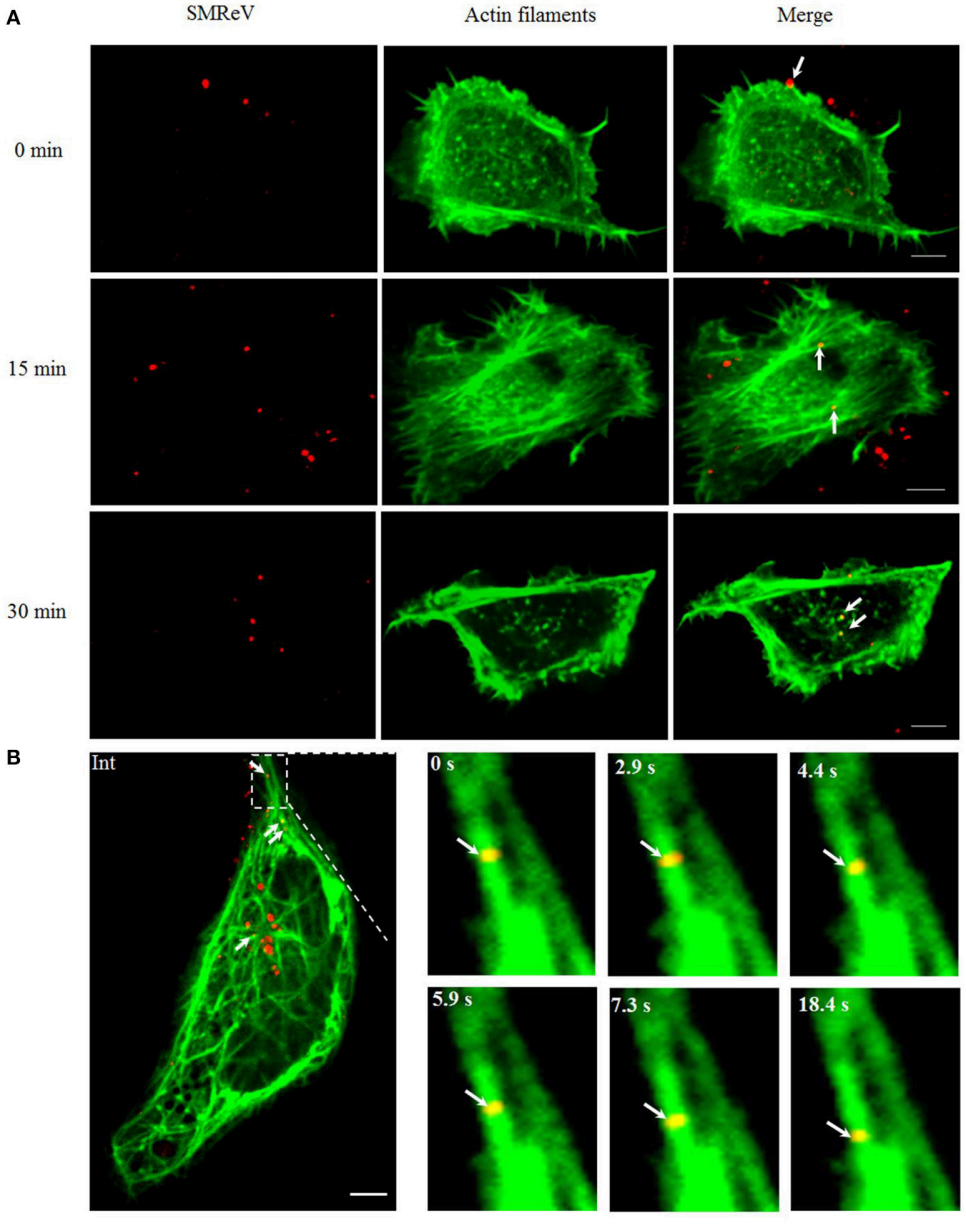
Cytoskeleton-dependent movement of SMReV particles in living GCF cells. **(A)** Time course of SMReV colocalization with actin filaments. QDs-SMReV particles (red) colocalized (yellow) with pRFP-LifeAct (green) labeling actin-rich protrusion at 0 min p.i. (0 min), with long actin filament near the cell periphery region at 15 min p.i. (15 min), and with dot-like actin at 30 min p.i. (30 min), respectively. **(B)** Snapshots of SMReV particles and microtubule. QDs-SMReV (Int, red) colocalized with pEGFP-MAP4 labeling microtubule (green) showing yellow signals (arrows mark). The enlargement images at different time (0–18.4 s) showed SMReV moved along the microtubule toward the cell interior. Bar: 5 μm.

Furthermore, we investigated whether the cytoskeletons are involved in SFTSV trafficking. Primarily, the correlation between SMReV particles and actin filaments was detected. The actin filaments recognized by pEGFP-LifeAct distributed mainly in the cell periphery. At 0 min postinfection, SMReV particle was located on the actin-rich protrusions extending from the cell surface (Figure 6A, 0 min), then SMReV particles colocalized with long actin filaments in the cell periphery region at 15 min (Figure 6A, 15 min). At 30 min, SMReV particles entered into the cell interior and just colocalized with dot-like actin (Figure 6A, 30 min). It was indicated that SMReV intracellular transport in the cell periphery was associated with actin filaments. In addition, single SMReV particle transport along the microtubule in living GCF cells was visualized in real-time, showing that SMReV particle retrograde transported along microtubule from the cell periphery to cell interior (Figure 6B).

**Figure 7:**
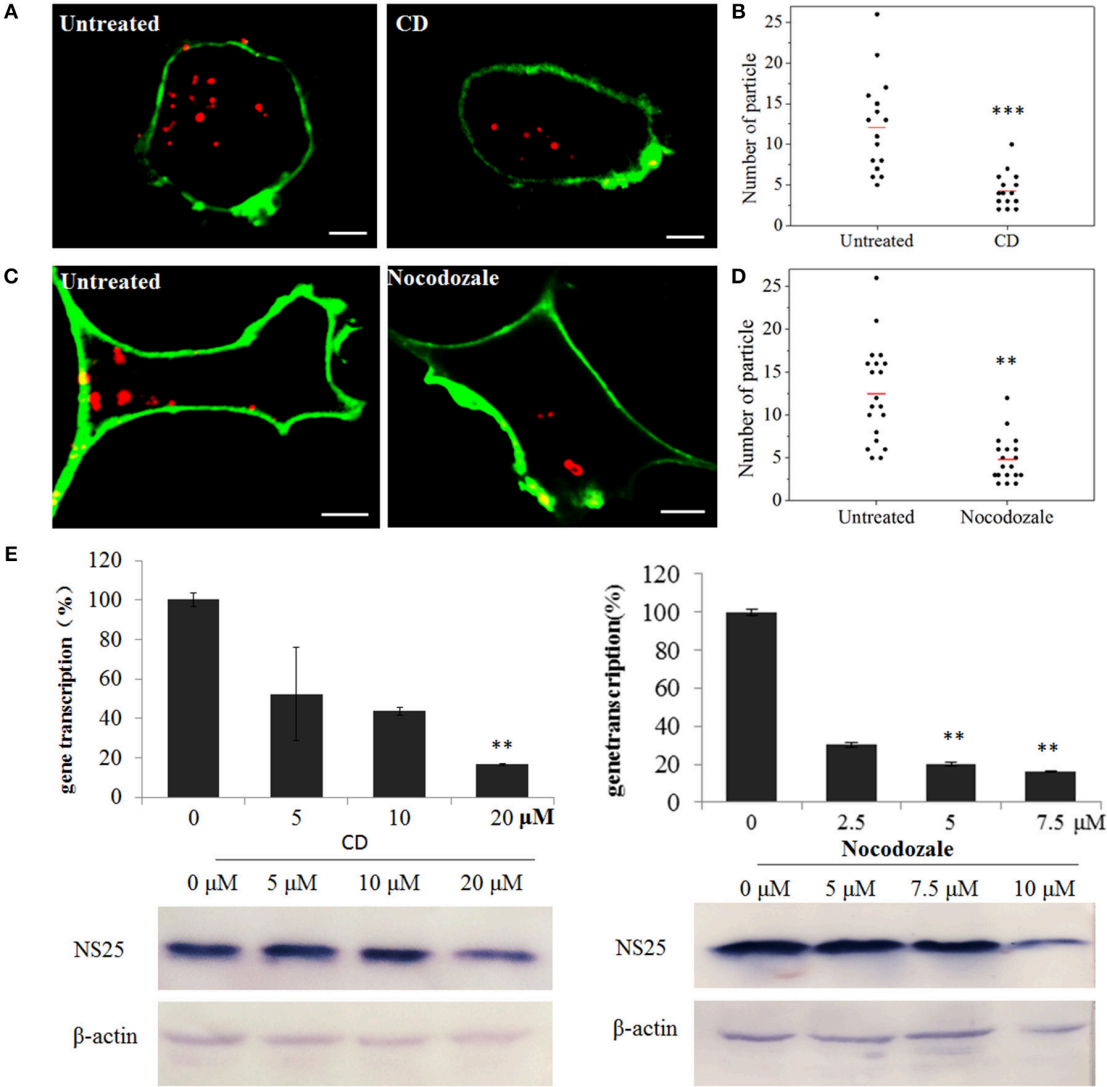
SMReV transport is dependent on actin filaments and microtubules. **(A,B)** Uptake of SMReV particles were reduced by CD treated. **(A)** Confocal images of QDs-SMReV (red) infected GCF cell untreated or treated with CD (10 μM) at 2 h p.i. The plasma membrane were labeled with CellMask (green). Bar: 5 μm. **(B)** Numbers of internalized QDs-SMReV particles in the cytoplasm of the untreated or treated cells. The bar showed the means of the data (*n* > 15 for each group). **(C,D)** Uptake of SMReV particles were reduced by nocodazole treated. **(C)** Confocal images of QDs-SMReV (red) infected GCF cell untreated or treated with nocodazole (7.5 μM) at 2 h p.i. The plasma membrane were labeled with CellMask (green). Bar: 5 μm. **(D)** Numbers of internalized QDs-SMReV particles in the cytoplasm of the untreated or treated cells. The bar showed the means of the data (*n* > 15 for each group). **(E)** Inhibitors of cytoskeleton reduced the SMReV infection. GCF cells were untreated (0 μM) or treated with CD (5–20 μM, left) or nocodazole (2.5–10 μM, right) for 2 h, infected with SMReV. Then the infected cells were collected at 72 h p.i. for gene transcription levels detection (upper panel) and NS25 protein expression analysis (lower panel). The viral gene transcription of untreated cells was factitiously set as 100%. The results are from triplicate experiments, and error bars indicate the means ± SD. ^*^p < 0.05, ^**^p < 0.01, ^***^p < 0.001. β-actin is used as a loading control.

To confirm the roles of microfilaments and microtubules in SMReV transport, the effects of CD (inhibits actin filament elongation at the barbed end) and nocodazole (microtubule-depolymerizing agent) on SMReV infection were detected. Compared with the controlled cell, the fluorescent signals of virus in the cytoplasm became less in the presence of CD or nocodazole (Figures 7A,C). Quantification of SMReV-infected cells (*n* > 15) showed that the number of internalized SMReV particles in CD or nocodazole treated cells was ~2.8- and 2.6-fold lower than in untreated cells, respectively (Figures 7B,D). Real-time qPCR analysis showed that the level of SMReV S8 gene transcription was decreased to 52, 43, and 16% of the control in the presence with 5, 10, 20 μM of CD, respectively, and decreased to 30, 20, and 15% of the control in the presence of increasing nocodazole concentrations (2.5–7.5 μM) (Figure 7E, upper panel). Western blotting assay also showed that expression of SMReV NS25 protein was lower in their host cells pretreated with CD (10 μM) or nocodazole (10 μM) (Figure 7E, lower panel). The data suggested that both actin filaments and microtubules were required for the cytoplasm motion of SMReV in host cell.

### Endosome-lysosome intracellular trafficking of the viral particles

**Figure 8:**
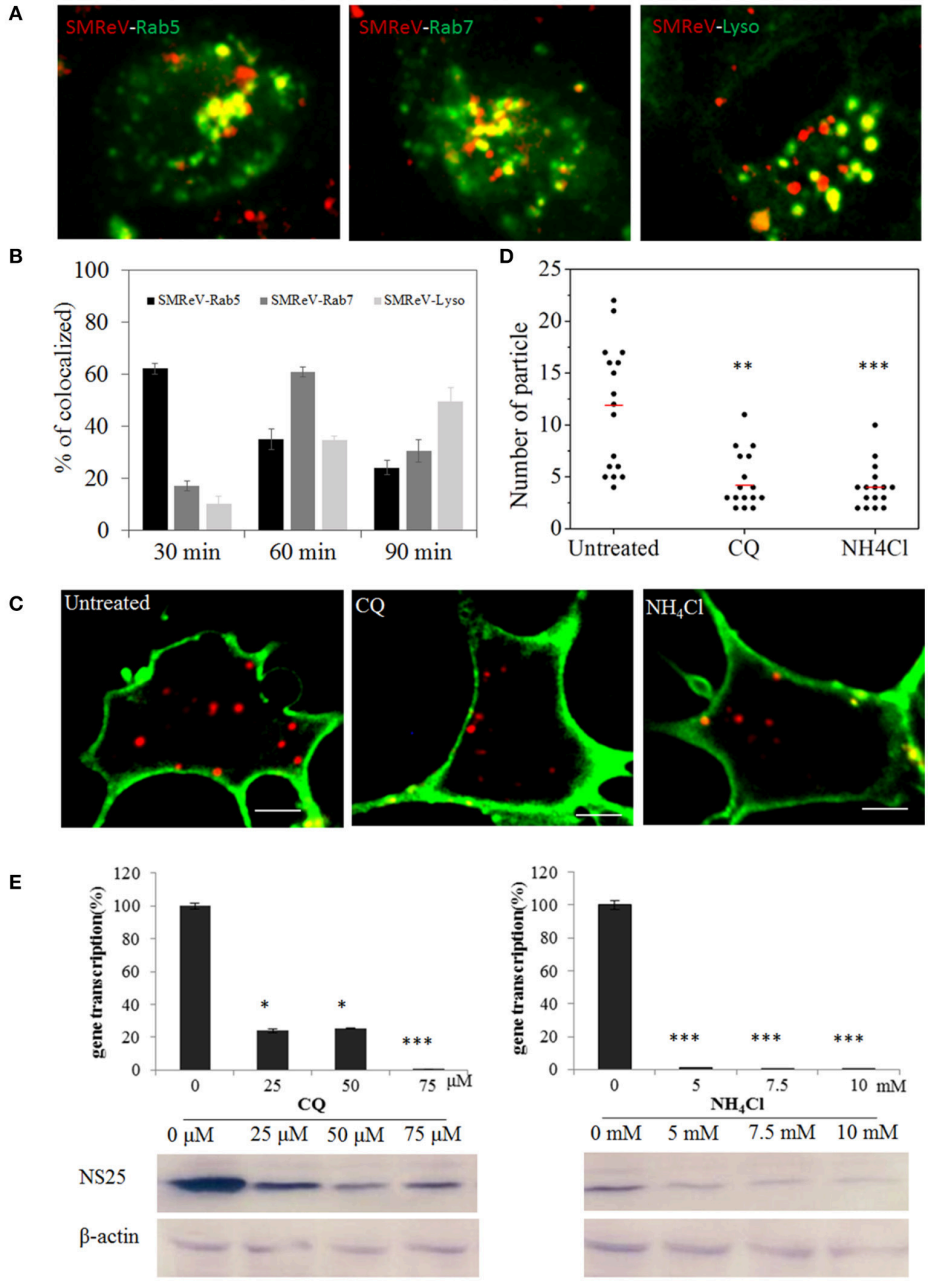
SMReV particles were trafficked through endosomes-lysosome system in GCF cytoplasm. **(A,B)** Time course of SMReV colocalization with endosomes-lysosome system. **(A)** Confocal images of QDs-SMReV particles and endosomes-lysosomes. particles mainly (red) colocalized (yellow) with pRFP-Rab5 labeling early endosomes (green) at 30 min p.i. (left), with pRFP-Rab7 labeling late endosomes (green) at 60 min p.i. (middle), and with LysoTracker labeling Lysosomes (Lyso) at 90 min p.i. (right), respectively. Bar: 5 μm. **(B)** Quantitation of colocalization of SMReV with Rab5, Rab7 or lysosomes at 30, 60, and 90 min p.i. (*n* ≥ 15 for each group). **(C,D)** Uptake of SMReV particles were reduced by CQ and NH_4_Cl. **(C)** Confocal images of QDs-SMReV (red) infected GCF cell untreated or treated with CQ (25 μM) and NH_4_Cl (5 mM) at 2 h p.i. The plasma membrane were labeled with CellMask (green). Bar: 5 μm. **(D)** Numbers of internalized QDs-SMReV particles in the cytoplasm of the untreated or treated cells. The bar showed the means of the data (*n* > 15 for each group). **(E)** Inhibitors of endosome-lysosomes reduced the SMReV infection. GCF cells were untreated (0 μM) or treated with CQ (0–75 μM, left) and NH_4_Cl (0–10 mM, right) for 2 h, infected with SMReV. Then the infected cells were collected at 72 h p.i. for gene transcription levels detection (upper panel) and NS25 protein expression analysis (lower panel). The viral gene transcription of untreated cells was factitiously set as 100%. The results are from two experiments, and error bars indicate the means ± SD. ^*^p < 0.05, ^**^p < 0.01, ^***^p < 0.001. β-actin is used as a loading control.

Once internalized into cells, virus then merged into the common endosomal network, and be sorted and disseminated to their final destinations. To determine if SMReV particles were tightly associated with the endosomes and lysosome, the colocation analysis of virus with endosomes-lysosome system were performed. The fluorescence images demonstrated that ~62, 35, and 24% of SMReV particles were colocalized with Rab5-positive (Rab5+) early endosomes at 30, 60, and 90 min postinfection, respectively; while 17, 60, and 30% of virions were colocalized with Rab7-positive (Rab7+) late endosomes at 30, 60, and 90 min postinfection, respectively; and 10, 35, and 49% of virions were colocalized with LysoTracker at 30, 60, and 90 min p.i., respectively (*n* ≥ 20 cells for each group) (Figures 8A,B). The results indicated that the delivery of SMReV in the cytoplasm involves sequential transfer from Rab5+ early endosomes to Rab7+ late endosomes, then to lysosomes. These endosomes were characterized by presence of low pH environments, which plays a vital role in viral penetration into the cytoplasm. Therefore, CQ and NH_4_Cl, weakly basic amines, were used to inhibit the acidification of endosomes and lysosome for determining the role of endosome-lysosome in the reovirus delivery. A fewer (~2.8- and 3-fold lower than the control, respectively) red fluorescent signals were detected in the pretreatment of cells with CQ (25 μM) and NH_4_Cl (5 mM) (Figures 8C,D), thus confirming the validity of the above assay. In the SMReV infected GCF cells, the levels of gene S8 transcription decreased to 24, 25, 0.5% of the controlled cells in the presence of 25, 50, and 75 μM CQ, respectively, and reduced to 0.9, 0.7, 0.5% of the untreated in the presence of increasing NH_4_Cl concentrations (0–75 μM) (Figure 8E, upper panel). Protein NS25 expression was also strongly inhibited by CQ (more than 25 μM) and NH_4_Cl (more than 5 mM), as shown in Figure 8E, lower panel. These result revealed that intracellular transport of SMReV was delivered *via* endosome–lysosome system.

## Discussion

Viral infection is a multi-step process involving many dynamic virus-host interactions. Although some critical entry steps of reoviuses have been investigated, the precise process and comprehensive picture of single virus particle entry is fuzzy or remained to be depicted (Schulz et al., 2012; Mainou et al., 2013; Wang et al., 2016; Zhang F. et al., 2018). Fluorescent labeling of virus particles and cellular structures simultaneously could made it possible to visualize the precise and dynamic process of virus entry and trafficking in host cells (Zhang et al. L. J., 2018). Here, we precisely described and visually tracing the entry and infection of reovirus, SMReV, using QDs-based single particle tracking combined with labeling cellular structures with gene maker, biochemistry analysis, and ultrastructural observation.

On the basis of the published labeling methods of QDs for enveloped virus (Liu et al., 2011; Hao et al., 2012; Zhang F. et al., 2013), we developed the labeling methods for non-enveloped virus by simplifying the purification approach of bio-SMReV and QDs-SMReV (Li et al., 2016). Through this, we could reduce the loss of the virus particles during the labeling process and obtain enough Bio-SMReV from once purification, which simplified the labeling approach of non-enveloped virus. And the TEM observation and infectivity analysis showed that QDs-SMReV particles still retain their native biological structure and infectivity. The image data obtained from QDs-SMReV was consistent with the results from biochemical assays and ultrastructural analysis by using native SMReV, so the QDs-SMReV can represent native SMReV to study the molecular mechanisms of viral infection. This improvement can promote the application of QDs in revealing the interaction between non-enveloped virus and host, providing a convenient tactic for exploring the life cycle of non-enveloped virus.

Different viruses applied various pathways to internalize into cells, even the same virus might enter diverse cells by several different routes (Wang et al., 2014; Andrade et al., 2017). Unlike the previous report (Li et al., 2016), in our study, the entry of SMReV was initiated with being embedded into the plasma membrane by the nascent clathrin-coat pit in 35.7 s, which is faster than invagination though the *de novo* assembly of clathrin invoveled in the enveloped virus (64–120 s) (Liu et al., 2011; Sun et al., 2017), and then the clathrin-coat pit containing fish reovirus pass through the membrane within 12.2 s (from 35.7 to 47.9 s). This was in accordance with the single particle analysis which showed membrane-bound SMReV could pass through the membrane within 14.8 s. And pretreatment with the inhibitor of the clathrin-mediated endocytosis significantly reduced viral infections, which prompt us that the viral entry pathway could be recognized as an ideal target for the design of antiviral strategies. These findings could be not only contributed to enrich our knowledge about the virus infection mechanisms, but also benefit the prevention and control of viral diseases in aquaculture.

Upon internalization, the viral particle confront the issue of how to reach the site of replication. Numerous viruses manipulate the host's cellular cytoskeleton system for efficient intracellular transportation Yang et al., 2018; Zhang et al. L. J., 2018). Precisely visualization and real-time tracing of single reovirus transport in the cytoplasm were carried out, and the intracellular transport routes and dynamics of non-enveloped virus was captured for the first time. The results show that reovirus moved slowly along the actin-enriched microfilament, while it moved relatively faster through the microtubules. A recent research shows that switchover between the motor proteins, myosin VI, and dynein, achieved the seamless transport of influenza viruses from actin filament at the cell periphery to microtubule during their infection (Zhang et al. L. J., 2018). In our study, we also observed this two kind of transport both involved in the SMReV intracellular transport. While whether the reovirus taking advantage of the same pattern to switch from the actin roadway to the microtubule highway remains obscure, or there are other more efficient methods. It still requires further research.

The reovirus not only used host's cytoskeleton-based transport system to benefit the movement, but also applied intracellular membrane trafficking pathways, such as using endosome-lysosome system for sorting and trafficking. Endosomes and lysosomes system are thought to play different roles in various cell processes, including collecting internalized cargoes, sorting, degradation and disseminating them to their final destinations (Scott et al., 2014). To release viral genome into the cytosol, enveloped viruses make use of membrane fusion in early endosomes or late endosomes (Lozach et al., 2011), while non-enveloped viruses undergo proteolytic disassembly to expose the viral membrane-penetration apparatus inducing membrane lysis or pore formation in endosomes-lysosomes (Gruenberg and van der Goot, 2006; Mainou and Dermody, 2012; Padilla-Parra et al., 2012). Late endosomes or lysosomes likely serve as disassembly sites for non-enveloped viruses, as these organelles are acidic and contain cathepsins. The intracellular compartment in which fish reovirus penetration occurs has not been identified. In our study, virus colocalization with early endosomes at earlier and with late endosomes at later were observed, then the particles displayed less colocalization with lysosomes. These results suggested that SMReV particles were sorted through early endosome to late endosome, finally disseminated to lysosome, in which the outer capsids of virus particles were disassembled by proteolytic cleavage. This disassembly may be significant for yielding metastable infectious subviral particle (ISVP) for productive infection (Zhang et al., 2006; Mainou and Dermody, 2011), otherwise may be due to the novel host defense mechanism of host to suppress virus infection through degradation to lead invalid infection (Chen et al., 2018).

## Conclusion

In the present study, the early infection journey of a non-enveloped virus, SMReV, have been investigated *via* QDs-based single particle tracking, biochemical analysis and ultrastructural observation. It was revealed that the reovirus particle could rapidly pass through the plasma membrane by ex-existed clathrin-caoted pits, subsequently transport along actin filament near the cell periphery and dependent on microtubule to the central cytoplasm. The intracellular particles were sorted from early endosome to late endosome, finally disseminated to lysosome for penetration. These findings shed light on the entry and intracellular dynamics interaction between host and the non-enveloped virus. Elucidating virus infection and initial steps is one of a major challenge for virology studies, and is of key importance to expand our knowledge of viral pathogenesis mechanism.

## Author contributions

JL and Q-YZ designed the experiments and wrote the paper. JL conducted the experiments. JL and CY analyzed the data. D-WP and J-FG edited and commented on the manuscript.

### Conflict of interest statement

The authors declare that the research was conducted in the absence of any commercial or financial relationships that could be construed as a potential conflict of interest.

## Supplementary material

Video S1 Visualization of SMReV internalization through clathrin-mediated endocytosis into GCF cells. QDs-SMReV particle (red) initially bound to a site near a pre-existing clathrin cluster (green), gradually got closely to and engulfed by clathrin cluster, then the clathrin cluster containing the particle pinched off the plasma membrane into the cytoplasm. The movie is played at 5 frames/s. See also corresponding Figure 3B.

Video S2 Visualization of SMReV transport in the host cell periphery region. QDs-SMReV (red) moved slowly along cell periphery. The movie is played at 10 frames/s. See also corresponding Figure 5A.

Video S3 Visualization of SMReV transport in the central cytoplasm. QDs-SMReV (red) moved relatively faster and oriented toward the central cytoplasm. The movie is played at 5 frames/s. See also corresponding Figure 5B.
